# Is angiogenesis a hallmark of prostate cancer?

**DOI:** 10.3389/fonc.2013.00015

**Published:** 2013-02-06

**Authors:** Gianluigi Taverna, Fabio Grizzi, Piergiuseppe Colombo, Pierpaolo Graziotti

**Affiliations:** ^1^Department of Urology, Humanitas Clinical and Research CenterRozzano, Milan, Italy; ^2^Laboratory of Molecular Gastroenterology, Humanitas Clinical and Research CenterRozzano, Milan, Italy; ^3^Department of Pathology, Humanitas Clinical and Research CenterRozzano, Milan, Italy

In vertebrate organogenesis, the blood vessels constitute the first organ system that arises and reaches a functional state (Persson and Buschmann, 2011). Angiogenesis, the development of new branching vessels from existing vasculature, is a complex process observed in fetal growth, wound healing and endometrial hyperplasia associated with the menstrual cycle (Carmeliet, 2003). Under these conditions, it is highly regulated: i.e., “turned on” for brief periods of time and then completely inhibited. However, many human diseases, including tumors, are driven by persistently up-regulated angiogenesis (Carmeliet, 2003, 2005; Hanahan and Weinberg, 2011). In some non-malignant diseases, such as lobular capillary hemangioma or keloid formation, angiogenesis is *self-limited*; however, this is not true of tumor angiogenesis, which, once begun, continues indefinitely until the entire tumor is eradicated or the host dies. Without blood vessels, tumors cannot grow beyond a critical size. Angiogenesis is regulated by a balance of pro- and anti-angiogenic molecules (Hanahan and Weinberg, 2011), secreted from cancer cells, endothelial cells and stromal cells (Fukumura et al., 1998), the relative contributions of which are likely to change with tumor type and site, as well as with tumor growth, regression and relapse. It is also ascertained that angiogenic vessels have a disorganized and irregular structure, and that the blood flow is abnormal. This is in contrast to the organized, regular structure and normal blood flow seen in mature vessels. Angiogenesis can be depicted as a non-linear dynamic process that is discontinuous in *space* and *time*, but advances through qualitatively different states. The term *state* defines the configuration pattern of the process at any given moment, and a dynamic process can be represented as a set of different states and a number of *transitions* from one state to another over a certain time interval. The *continuum* of these states generates a complex ramified structure that irregularly fills the surrounding environment. The main feature of the newly generated vasculature is the structural diversity of the vessel sizes, shapes, and connecting patterns. This is mainly due to the heterogeneous distribution of angiogenic regulators, such as vascular-endothelial growth factor, basic fibroblastic growth factor and angiopoietin, leading to hypoxic and acidic tumoral regions (Karlou et al., 2010). Moreover, although it is commonly believed that the endothelial cells making-up tumor vessels are genetically stable, tumor vasculature seems to be much more unpredictable (Streubel et al., 2004). These conditions all reduce the effectiveness of treatments, modulate the production of pro- and anti-angiogenic molecules, and select a subset of more aggressive cancer cells with higher metastatic potential.

The significance of angiogenesis in prostate cancer (PC) still remains controversial (Russo et al., 2012). While there are currently no markers of the net angiogenic activity of PC that can help investigators to design specific anti-angiogenic treatment strategies, it is reasonable to assume that the quantification of various aspects of tumor vasculature may provide an indication of angiogenic activity. One often-quantified parameter of PC vasculature is microvessel density (MVD), which is used to allow a histological assessment of tumor angiogenesis. The results of studies carried out over the last decade have suggested the value of using MVD as a prognostic index in PC, and it has also assumed that MVD may reveal the degree of angiogenic activity in PC. MVD has, however, a number of limitations. The conflicting MVD results in PC are likely due to the differences in study designs: variability in patient population size, tumor topography, approach to selection of representative tumor areas, choice of endothelial marker, and actual counting method. The selection of the tumor area for MVD assessment has been based on two different approaches: (1) analysis of a few microscopic “hot spots” containing the maximal vascular density, and (2) selection of random representative areas of the tumor. The first approach is the most applied due to its simplicity, although there is no agreement among investigators regarding optimal microscope magnification, the number of vascular hot spots, and cutoff values for low vs. high MVD. The second approach of MVD assessment within larger representative areas or whole tissue may be more objective but involves more tedious examination.

Despite its importance as a prognostic indicator in untreated tumors, MVD has not been shown to be a valid measure to guide or evaluate anti-angiogenic treatment (Hlatky et al., 2002). MVD does not appear to be predictive of tumor response under anti-angiogenic treatment and therefore may not be useful for stratifying patients for clinical trials (Rubin et al., 1999; Eberhard et al., 2000; Hlatky et al., 2002; Preusser et al., 2006; Erbersdobler et al., 2010). Low MVD does not portend a poor response to anti-angiogenic therapy (Vartanian and Weidner, 1995; Eberhard et al., 2000; Hlatky et al., 2002). Tumor MVD may not vary in accordance with the tissue or blood levels of any single pro-angiogenic factor. Rapid tumor growth does not imply high MVD. The MVD of a tumor need not be higher, and is often lower, than that of its corresponding normal tissue, which is experiencing no net growth. The efficacy of anti-angiogenic agents cannot be simply visualized by alterations in MVD during treatment (Pluda, 1997; Hlatky et al., 2002). In addition, MVD is substantially limited by the complex biology characterizing tumor vasculature (Aird, 2012), and the highly irregular geometry that the vascular system assumes in real space, which cannot be measured using the principles of Euclidean geometry because it is only capable of interpreting regular and smooth objects that are almost impossible to find in Nature (Grizzi et al., 2007). Quantitative descriptors of its geometrical complexity can be, however, abstracted from the Fractal geometry introduced by Benoit Mandelbrot in 1975 (Baish and Jain, 2000; Grizzi et al., 2005). The complex geometry of tumor vasculature and its structural and functional heterogeneity mean that vascular network cannot be measured on the basis of MVD estimates alone. Tetiakova et al. applying an automated image analysis to conventional and tissue microarray sections in large representative areas demonstrated that there was no significant increase in MVD parameters in PC vs. matched normal peripheral zone prostatic tissue (Tretiakova et al., 2012). Paradoxically, several morphological indexes were higher within normal glandular prostatic tissue. Deering et al. also reported no increase in MVD counts between benign prostatic hyperplasia and PC by computer-assisted image analysis (Deering et al., 1995). Barth et al. demonstrated that direct stereologic assessment of vascular surface density quantitating the vessel area per tissue volume showed no significant difference between normal and PC tissue from grade 2 and grade 3 tumors (Barth et al., 1996). Another study of two-dimensional vascularity of PC by Taverna et al. (2009) divided all cases in two groups with 56% of cases showing increase of vascular surface in PC vs. non-tumoral areas and 44% showing a decrease of vascular surface in PC. The second group of patients with lower tumoral vascular surface had a poorer outcome indicating that tumor progression is independent of angiogenesis (Taverna et al., 2009). These findings parallel recent data by Steiner et al. (2012) who showed no significant difference for CD31 mRNA levels from normal prostate and matched PC (*P* = 0.78). No significant correlation for CD31 between mRNA and protein levels by immunohistochemistry implies that in the typical slow growth of PC, the angiogenesis dynamics are also quite low (Steiner et al., 2012).

A non-invasive imaging technique that could reflect MVD would hold great promise in tumor detection and characterization (Jain et al., 1997; Jiang et al., 2011). An imaging method that could indicate an increase in MVD could have value in choosing targets for prostate biopsies (Jiang et al., 2011). This will lead to a change in biopsy strategies, bringing about a higher detection rate of PC, and hence, a more appropriate therapeutic strategy (Jiang et al., 2011). Preliminary data suggested that the haemodynamic indices obtained from contrast-enhanced ultrasound imaging were different between low- and high-grade PCs (Jiang et al., 2011). The microvessels that proliferate in PC are below the resolution of conventional Doppler imaging; only the larger vessels are visualized by this imaging technique (Halpern, 2006). Franiel et al. attempt to determine whether established histologic parameters of prognostic importance, including MVD, correlate with parameters obtained at pharmacokinetic dynamic contrast material–enhanced (DCE) dual-contrast-enhanced magnetic resonance (MR) imaging (Franiel et al., 2009). They found that blood volume and interstitial volume did not reliably correlate with the histologic parameters, mainly due to the heterogeneous vascularization of both normal prostate tissue and PC (Mucci et al., 2009). Variability over patients is large with patients showing both increased and decreased vascularity in the tumor (van Niekerk et al., 2012). Thus, determination of vascularization in a two-dimensional histological slide is not representative of the vascularity of the tissue as a whole (Rubin et al., 1999; Grizzi et al., 2005). The antibody used also seems to play a role, since it has been shown that MVD immunohistochemically determined with CD31 antibody staining was significantly lower than that obtained with CD34 antibody staining (De La Taille et al., 2000). Moreover, correlation of histologic and MR data sets was limited by the fact that the paraffin sections are 4 μm-tick, whereas the corresponding T2-weighted images have a slice thickness of 3 mm and the dynamic susceptibility weighted MR DCE-MR sequence is acquired with a slice thickness of 5 mm (Franiel et al., 2010). Computer-based 3D prostate models may in the future enable the desired detail correlation between histologic and MR imaging findings (Franiel et al., 2010). The lack of correlation between histologic and functional parameters also raises the question of the biologic significance of functional parameters of tumor microcirculation quantified with dynamic imaging enhanced with small-molecule contrast medium (Cyran et al., 2012). Although, Osimani et al. have recently shown that blood volume and permeability surface-area product measurements obtained with perfusion computed tomography have the highest correlation with immunohistochemical markers of angiogenesis in PC, before routine implementation, additional studies on larger series are needed (Osimani et al., 2012).

Scientific knowledge develops through the evolution of new concepts, and this process is usually driven by new methodologies that provide previously unavailable observation. The potential broad applicability of the Fractal geometry makes it possible to explore the range of the morphological variability of neovasculature that can be produced in nature, thus increasing its diagnostic importance in cancer research (Taverna et al., 2010; Steiner et al., 2012). Prostate histology may remain the reference method for assessing the *status* of neovascularity, but there are still several open questions, including whether angiogenesis is a canonic hallmark of PC, and the absence of a powerful method of quantifying the reversal of neovascularity.
